# Screening of a Novel Polysaccharide Lyase Family 10 Pectate Lyase from *Paenibacillus polymyxa* KF-1: Cloning, Expression and Characterization

**DOI:** 10.3390/molecules23112774

**Published:** 2018-10-26

**Authors:** Yan Zhao, Ye Yuan, Xinyu Zhang, Yumei Li, Qiang Li, Yifa Zhou, Juan Gao

**Affiliations:** 1 School of Biological Science and Technology, University of Jinan, Jinan 250022, China; zhaoyan_1994@126.com (Y.Z.); zhangxinyu950512@163.com (X.Z.); mls_liym@ujn.edu.cn (Y.L.); chm_liq@ujn.edu.cn (Q.L.); 2 School of Life Sciences, Northeast Normal University, Changchun 130024, China; yuany268@nenu.edu.cn (Y.Y.); zhouyf383@nenu.edu.cn (Y.Z.)

**Keywords:** *Paenibacillus polymyxa*, pectate lyase, cloning and expression, ramie degumming

## Abstract

Pectate lyase (EC 4.2.2.2) catalyzes the cleavage of α-1,4-glycosidic bonds of pectin polymers, and it has potential uses in the textile industry. In this study, a novel pectate lyase belonging to polysaccharide lyase family 10 was screened from the secreted enzyme extract of *Paenibacillus polymyxa* KF-1 and identified by liquid chromatography-MS/MS. The gene was cloned from *P. polymyxa* KF-1 genomic DNA and expressed in *Escherichia coli*. The recombinant enzyme PpPel10a had a predicted Mr of 45.2 kDa and *p*I of 9.41. Using polygalacturonic acid (PGA) as substrate, the optimal conditions for PpPel10a reaction were determined to be 50 °C and pH 9.0, respectively. The K_m_, v_max_ and k_cat_ values of PpPel10a with PGA as substrate were 0.12 g/L, 289 μmol/min/mg, and 202.3 s^−1^, respectively. Recombinant PpPel10a degraded citrus pectin, producing unsaturated mono- and oligogalacturonic acids. PpPel10a reduced the viscosity of PGA, and weight loss of ramie (*Boehmeria nivea*) fibers was observed after treatment with the enzyme alone (22.5%) or the enzyme in combination with alkali (26.3%). This enzyme has potential for use in plant fiber processing.

## 1. Introduction

Pectin is a heteropolysaccharide mainly composed of α-1,4-linked galacturonic acids with different degrees of esterification [1,2]. Pectin is widely present in the cell walls of terrestrial plants and has a wide range of applications in industries such as food, medicine and fine chemicals [1,3]. Recent studies showed that the degradation products of pectin have good physicochemical properties, and wide application prospects as food additives, pharmaceutical molecules, and materials for cosmetics [4,5].

The complete degradation of pectin requires the synergistic work of a series of enzymes, including pectate lyase, pectin methylesterase, and polygalacturonase [2,6]. Pectate lyases (EC 4.2.2.2), also called trans-eliminases, catalyze the cleavage of pectate via a β-elimination reaction to generate 4,5-unsaturated oligogalacturonates [7,8]. Recently, pectate lyase has received increasing attention because of its potential applications in various industries, such as in the extraction and clarification of fruit juices and wine, scouring of cotton fabric, retting of flax, degumming of ramie fibers, and treatment of pectic wastewater [7].

Ramie (*Boehmeria nivea*) fibers have wide application potential, and can be used to make clothing fabrics, twines and canvas [9]. The surface of ramie fiber is covered with gum-like materials, which limit its application in the textile industry—The gum-like materials must be removed before application. The traditional degumming process is performed by alkaline treatment at high temperature, which damages the fibers and causes environmental pollution. However, the degumming could also be achieved by enzymatic catalysis performed by pectate lyase [10,11]. Enzymatic degumming has advantages compared with alkaline degumming because of its high efficiency, mild conditions and environmental compatibility.

According to the pH at which they show maximal activity, pectate lyases can be divided into acidic and alkaline groups. Alkaline pectate lyases are preferred for degumming ramie since pectin is more soluble in alkaline solution [12]. Alkaline pectate lyases have been isolated from various microbial sources, including bacteria and fungi [13]. The genus *Bacillus* is one of the main sources of pectic lyase [11,13,14,15,16]. In this study, *Paenibacillus polymyxa* KF-1 (also known as *Bacillus polymyxa*) was found to exhibit remarkable pectate lyase activity. A pectate lyase was identified from the enzyme extract of *P. polymyxa* KF-1 and expressed in *Escherichia coli*. The recombinant enzyme was purified, and its ability to remove the pectin from ramie was explored.

## 2. Results and Discussion

### 2.1. Screening of Pectate Lyase from P. polymyxa KF-1 by LC-MS/MS

**Figure 1:**
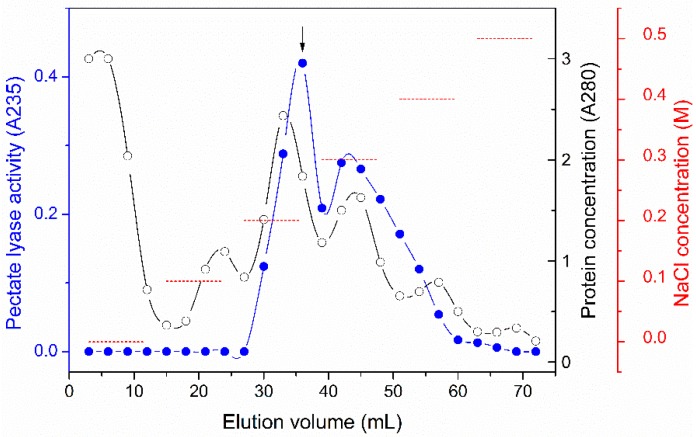
Elution profile of pectate lyase from *P. polymyxa* KF-1 culture broth by SP Sepharose fast-flow column chromatography. (-●-) Pectate lyase activity (A235); (-○-) protein concentration determined by absorbance at 280 nm (A280); (----) NaCl concentration. The arrow indicates the fraction used for LC-MS/MS analysis.

Genes encoding pectate lyases have been cloned from *Bacillus* spp., such as for the pectate lyase from *B. tequilensis* SV11; PEL168, Apel and rePelB from *B. subtilis*; BliPelA from *B. licheniformis*; Bsp165PelA from *Bacillus* spp. N16-5; Pel-15E from *Bacillus* spp. strain KSM-P15; and Pel SWU from *Bacillus* spp. RN1 [13,14,15,16,17,18]. Here, *P. polymyxa* exhibited pectin-degradation activity. Using citrus pectin as the substrate, the pectate lyase activity accumulated with prolonged cultivation time, up to 36 h (Figure S1). These results suggested that *P. polymyxa* is a good candidate to screen for pectate lyase. The supernatant of *P. polymyxa* KF-1 culture (i.e., the fermentation broth) was purified by ion exchange chromatography performed using an SP Sepharose fast-flow column. Two peaks with pectate lyase activity were observed in the elution profile (Figure 1); the fraction eluted by 0.2 M NaCl (fraction 12) exhibited the highest pectate lyase activity and was sent for LC-MS/MS analysis. A total of 28 proteins were identified from the faction, including four pectate lyases, one pectin esterase and 23 proteins which were not pectin-degrading enzymes. As shown in Table 1, ten peptides were retrieved and assigned to four pectate lyases, which belonged to PL families 1, 3, 9, and 10, respectively, predicted using the CAZy database (http://www.cazy.org/) [19]. The amino acid sequence of the protein with UniProt accession number E3EEN8 (NCBI protein ID WP_013370345.1) was consistent with a PL family 10 pectate lyase. The PL10 enzyme showed the highest intensity in LC-MS/MS analysis (Table 1), which suggested that the enzyme may be a major component of the pectin-degrading enzymes of *P. polymyxa* KF-1. Only five pectate lyases belonging to PL family 10 have been characterized, including PelA from *B. alcalophillus* NTT33 [20], Pel-15E from *Bacillus* spp. strain KSM-P15 [21], PelA from *Azospirillum irakense* [22], rPelA from *Treponema pectinovorum* ATCC 33768 [23], and r-PL_D from *Xanthomonas campestris* ACCC 10048 [24]. Reported PL10 enzymes had different structure from the reported members in PL family 1, 3, and 9, and were active at alkaline conditions, which may be potential in textile industry [20,21,22,23,24]. However, the properties especially the substrate specificity of PL10 enzymes are still unclear. The application of PL10 enzymes on ramie degumming have not been studied. Understanding of PL family 10 pectate lyases are needed for their application. Thus, the gene encoding the PL family 10 enzyme from *P. polymyxa* KF-1 was chosen for cloning and expression in the present study.

### 2.2. Gene Cloning and Sequence Analysis

**Figure 2:**
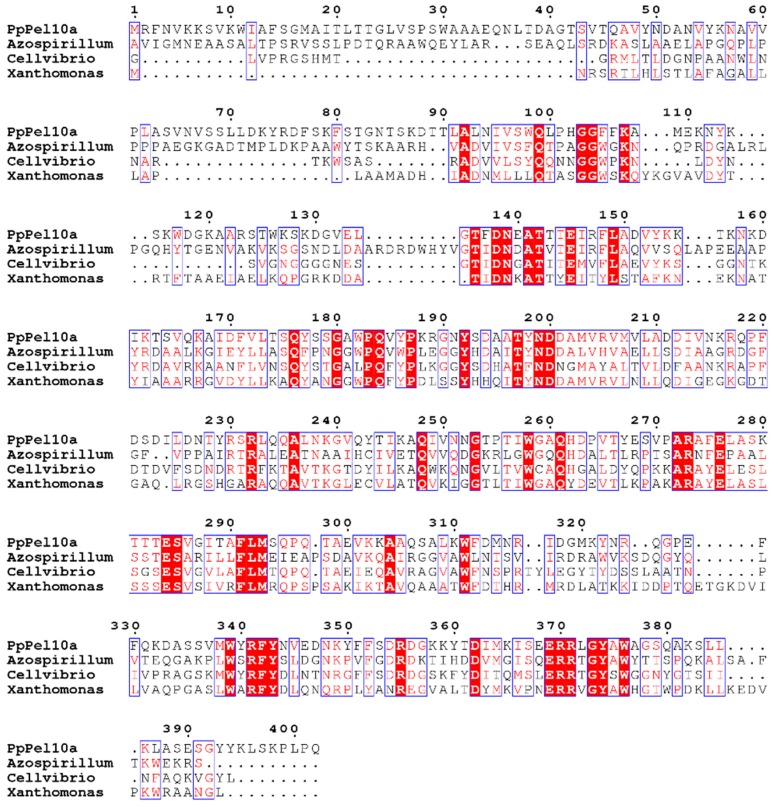
Alignment of amino acid sequence of PpPel10a with those of PL10 family pectate lyases from *C. cellulosa* (PDB ID: 1GXN), *A. irakense* (AF121904), and *X. campestris* (JQ723690).

Analysis of the *P. polymyxa* genome revealed six genes which were predicted to encode pectate lyases belonging to PL family 1, 3, 9, and 10, respectively. The accession numbers in NCBI for the genes were WP_013372506.1, WP_014599567.1, WP_013369567.1, WP_013370345.1, WP_013373703.1, and WP_013308307.1. Only one PL10 enzyme was retrieved. The gene encoding pectate lyase from *P. polymyxa* KF-1 was cloned. The open reading frame of 1209 bp encoded a protein of 402 amino acids. The N-terminal 33 amino acids were predicted to be signal peptide by the SignalP 4.1 server. The predicted Mr/*p*I values were 45.24 kDa and pH 9.41. Generally, pectate lyases are classified into PL families 1, 2, 3, 9, and 10 according to the CAZy database [19]. The amino acid sequence of PpPel10a from *P. polymyxa* KF-1 showed similarity to the characterized PL family 10 pectate lyases (e.g., AF121904.1, 35%; JQ723690, 40%) (Figure 2). Using the family 10 polysaccharide lyase from *Cellvibrio cellulosa* (PDB ID: 1GXN, identity = 44.79%) as a template [27], the structure of PpPel10a was modelled; it displayed a predominantly α-helical structure with short β-strands and irregular coils (Figure S2). The structure of family 10 pectate lyases is different from that of the pectate lyases from PL families 1, 3 and 9, which have a parallel β-helix fold [28]. From the amino acid sequence alignment it is deduced that residues D138, N139, R273, E276, R355 and R370 of PpPel10a were responsible for catalysis.

### 2.3. Expression of Recombinant PpPel10a and Enzyme Purification

The gene encoding the mature pectate lyase PpPel10a was expressed in *E. coli* BL21 (DE3) cells. After induction with 0.5 mM isopropyl β-d-1-thiogalactopyranoside (IPTG) at 25 °C for 10 h, pectate lyase activity was detected mainly in the cell lysate, suggesting the soluble expression of recombinant PpPel10a. Recombinant PpPel10a was purified by Ni-NTA column chromatography, eluted by 50 mM imidazole.

The specific activity of purified enzyme was determined to be 290 U/mg (Table 2), which was in the range of reported microbial pectate lyases (45–600 U/mg) [10,11,12]. Using polygalacturonic acid (PGA) as substrate, the K_m_ and v_max_ values for the enzyme were determined to be 0.12 g/L and 289 μmol/min/mg, respectively. The k_cat_ was calculated to be 202.3 s^−1^. 

**Figure 3:**
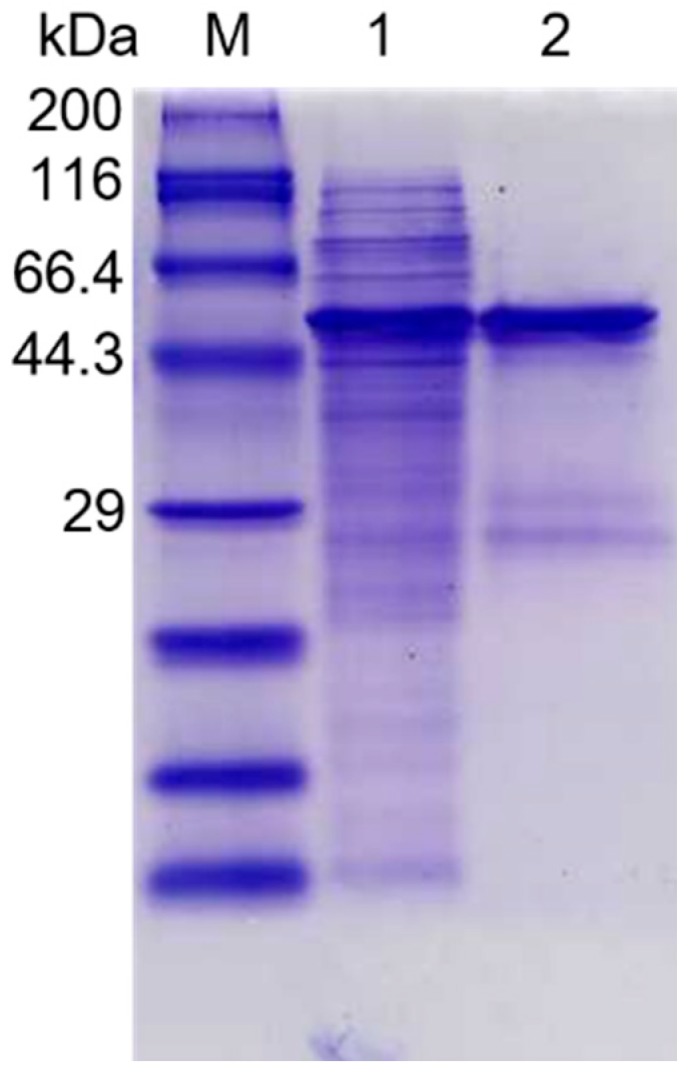
SDS-PAGE analysis of recombinant PpPel10a. M, molecular weight markers; lane 1, supernatant of lysed recombinant *E. coli* BL21 (DE3) cells carrying plasmid pET28a-pppel10a; lane 2, PpPel10a purified by Ni-NTA column chromatography.

The molecular weight of purified recombinant PpPel10a was determined to be approximately 45.2 kDa by SDS-PAGE (Figure 3), which was similar to reported values for PL10 enzymes, such as PelA from *B. alcalophillus* NTT33 (~35 kDa), Pel-15E from *Bacillus* spp. strain KSM-P15 (~33 kDa), PelA from *A. irakense* (44.5 kDa), rPelA from *T. pectinovorum* ATCC 33768 (~49 kDa), and r-PL D from *X. campestris* ACCC 10048 (~38 kDa) [20,21,22,23,24].

### 2.4. Enzymatic Properties of Purified PpPel10a

**Figure 4:**
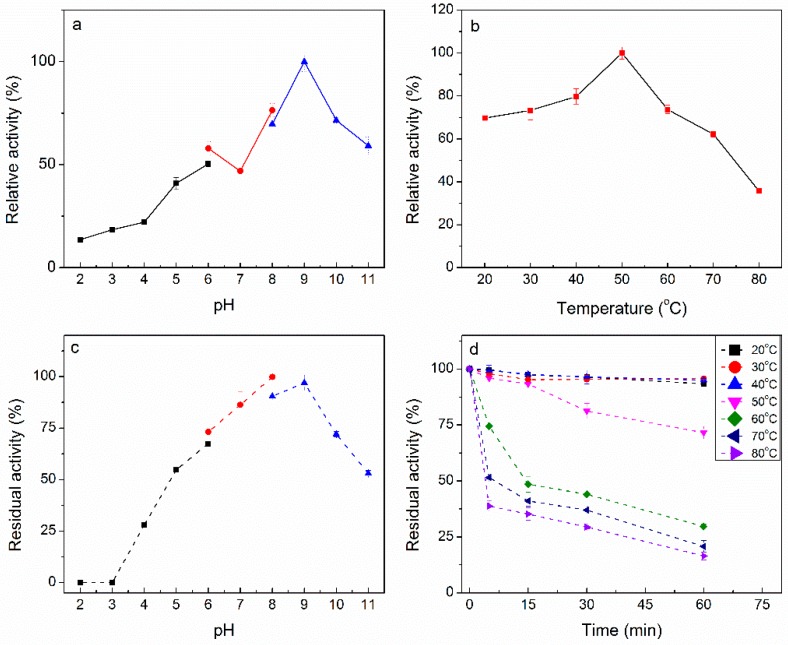
Effect of pH and temperature on activity and stability of PpPel10a. (**a**) Optimal reaction pH, (-■-) pH 2.0–6.0, 50 mM sodium acetate buffer; (-●-) pH 6.0–8.0, 50 mM phosphate buffer; (-▲-) pH 8.0–11.0, 50 mM sodium glycine buffer; (**b**) optimal reaction temperature; (**c**) pH stability, (-■-) pH 2.0–6.0, 50 mM sodium acetate buffer; (-●-) pH 6.0–8.0, 50 mM phosphate buffer; (-▲-) pH 8.0–11.0, 50 mM sodium glycine buffer; (**d**) thermostability. Pectate lyase activity was measured using polygalacturonic acid (PGA) as substrate.

The optimal pH for activity of PpPel10a was determined to be pH 9.0; the enzyme was very active over a wide pH range (≥70% of maximum activity at pH 7.0–11.0) (Figure 4a). Alkaline pectate lyases have mainly been cloned from *Streptomyces* and *Bacillus* spp. [24]. The optimal pH of PpPel10a was similar to that of Apel from *B. subtilis* (pH 9.0) and r-PL-STR from *Streptomyces* sp. S27 (pH 9.5), but higher than that of Pel from *B. subtilis* 168 (pH 8.0) [15,21,29]. PpPel10a showed stability over a wide pH range; the enzyme retained >50% of its initial activity after being held at pH 5.0–11.0 for 24 h (Figure 4b). The pH stability of PpPel10a was broader than those of the pectate lyases PEL168 from *B. subtilis* 168 (pH 7.0–9.5), Apel from *B. subtilis* (pH 7.0–10.0), and r-PL-STR from *Streptomyces* sp. S27 (pH 7.0–10.0) [13,15,29]. So far, only one PL10 enzyme r-PL D from *X. campestris* ACCC 10048 was characterized on its pH stability [24]. r-PL D was stable over a broad pH range, retaining more than 50 % of its initial activity after incubation at pH 3.0–12.0. PpPel10a showed similar alkaline-tolerance property as r-PL D, which indicated that alkaline-tolerance may in common among PL10 enzymes. The optimal temperature for activity of PpPel10a was 50 °C (Figure 4c), which was consistent with that of Apel from *B. subtilis* (50 °C), but lower than those of PelA from *B. licheniformis* 14A (70 °C), and BacPelA from *B. clausii* (70 °C) [14,30]. PpPel10a was quite stable at <50 °C, but lost >50% of its initial activity after incubation at 60 °C for 30 min (Figure 4d). These remarkable alkaline-active, alkali-stable and thermostable properties make PpPel10a an excellent candidate for application in industries requiring alkaline reaction conditions.

**Figure 5:**
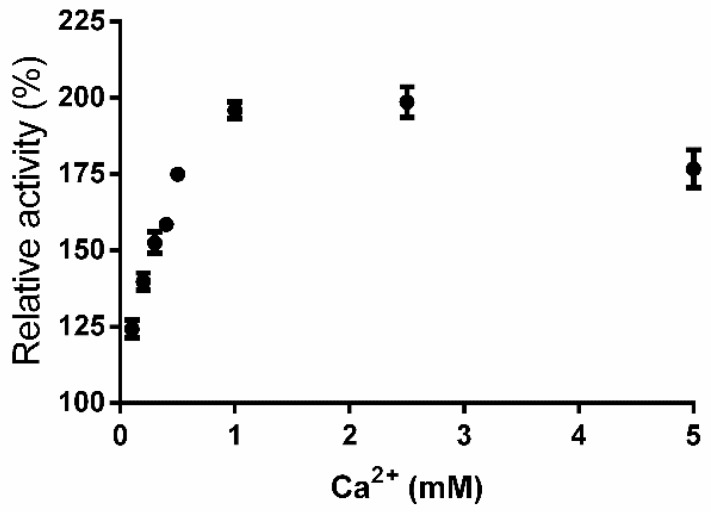
Effect of Ca^2+^ addition on the activity of PpPel10a.

PpPel10a exhibited remarkable tolerance to metal ions and chemical reagents. As Table 3 shows, Fe^2+^, Co^2+^, SDS and Triton X-100 were strong inhibitors of enzymatic activity at both 1 and 5 mM. Ca^2+^ was reported to be essential for the activity of most pectate lyases (optimal Ca^2+^ concentration in the range 0.1 to 1.0 mM). The enzymatic activity of PpPel10a was enhanced by the addition of Ca^2+^; a dose-dependent effect was observed at 0.1–2.5 mM [31]. The presence of 2.5 mM Ca^2+^ enhanced the enzyme activity by 1.98-fold compared with that in the absence of added Ca^2+^ (Figure 5). Therefore, the addition of 2.5 mM Ca^2+^ was employed in further experiments. Mg^2+^, Zn^2+^ and Al^3+^ at 1 mM slightly enhanced the enzymatic activity, while other metal ions and chemicals tested showed no significant effect on the enzymatic activity at 1 or 5 mM.

### 2.5. Identification of Products from Citrus Pectin on Degradation by PpPel10a

**Figure 6:**
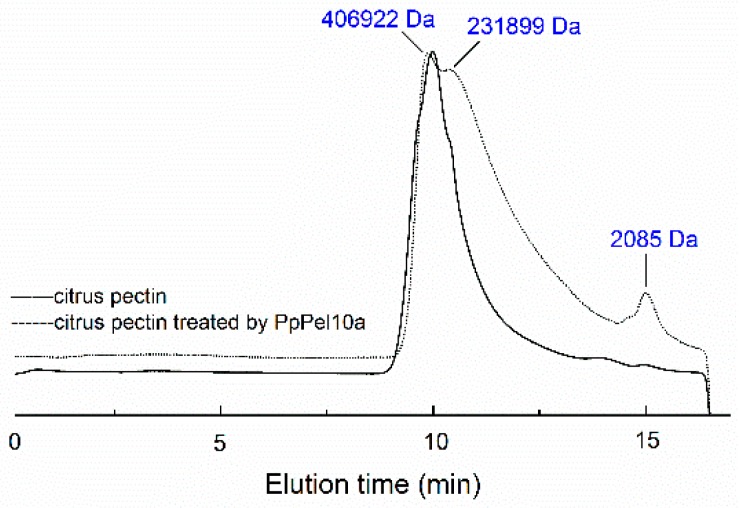
High-performance gel permeation chromatography analysis of the lytic products of citrus pectin produced by PpPel10a. (—) Citrus pectin; (-----) lytic products of citrus pectin.

**Figure 7:**
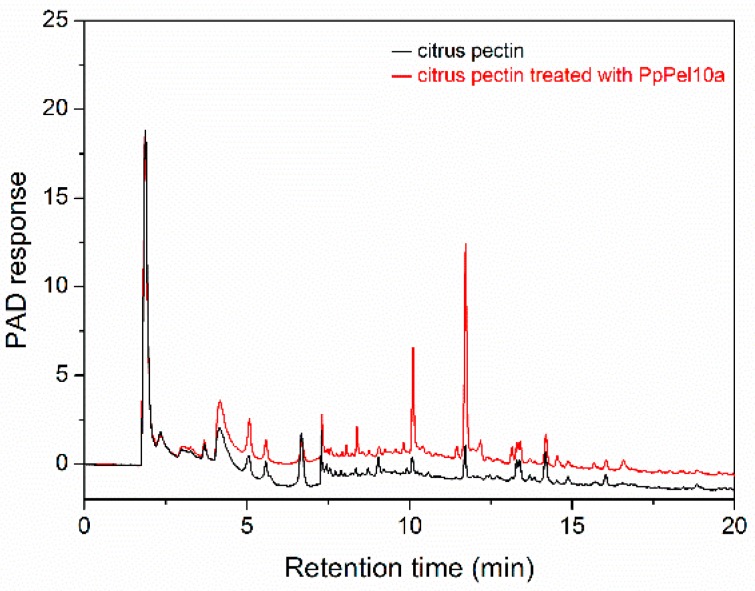
High-performance anion exchange chromatography analysis of the lytic products of citrus pectin produced by PpPel10a. Black line, citrus pectin; red line, lytic products of citrus pectin.

High-performance gel permeation chromatography (HPGPC) analysis showed that citrus pectin (~406,922 Da) was degraded to smaller molecular weight species (~231,899 Da and ~2085 Da) by PpPel10a (Figure 6). The oligomers released from citrus pectin by PpPel10a were observed on high-performance anion exchange chromatography (HPAEC) (Figure 7). 

**Figure 8:**
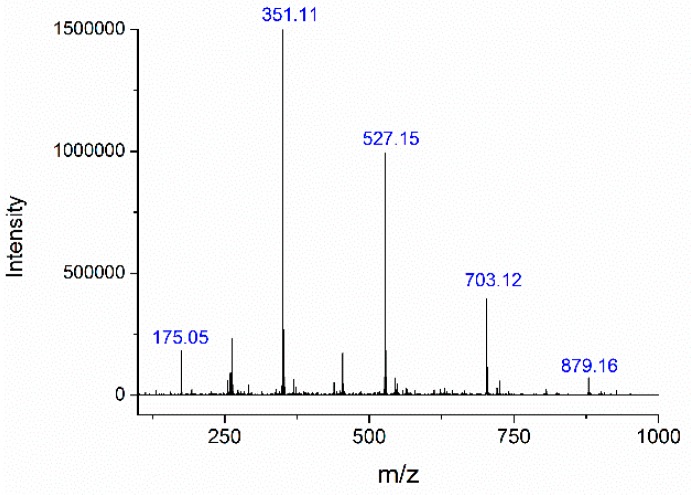
Electrospray ionization-mass spectrometry analysis of the lytic products of citrus pectin produced by PpPel10a.

The oligosaccharides were identified by ESI-MS. Negative ESI-MS gave strong peaks at *m*/*z* 175 (unsaturated galacturonic acid, uG1), *m*/*z* 351 (unsaturated bigalacturonide, uG2), *m*/*z* 527 (unsaturated trigalacturonide, uG3), *m*/*z* 703 (unsaturated tetragalacturonide, uG4), and *m*/*z* 879 (unsaturated pentagalacturonide, uG5) (Figure 8) [16]. The abundance was in the order uG2 > uG3 > uG4 > uG1 > uG5. These results indicate that citrus pectin was degraded by PpPel10a, producing a mixture of 4,5-unsaturated monogalacturonic acid and oligogalacturonic acid, confirming the trans-elimination reaction mechanism of PpPel10a. Generally, exo-acting pectate lyase produces only monomer or dimer, while endo-acting pectate lyase releases larger oligomers [16]. The HPAEC and ESI-MS results showed that uG2 and uG3 were the main products released from citrus pectin by PpPel10a, indicating PpPel10a is an endo-acting enzyme. The catalytic behavior of PpPel10a was similar to that of Pel SWU, the PL family 1 endo-acting enzyme from *Bacillus* spp. RN1, but different from some other reported endo-acting pectate lyases, such as PelA from *B. licheniformis* 14A and PL from *B. subtilis*, which produced unsaturated oligogalacturonides uG2 and uG3, but not uG1 [14,15,16]. Only two PL10 enzymes have been reported on their catalytic behavior. PelA from *A. irakense* was an endo-acting pectate lyase, producing multiple unsaturated oligogalacturonates (uG2 to uG9) without the accumulation of uG1 [22]. r-PL D from *X. campestris* ACCC 10048 was an exo-type pectate lyase, the major product was determined to be uG2 [24]. Thus, to our knowledge, this is the first report of a PL10 enzyme that can accumulate uG1 from pectin. It is deduced that PL10 enzymes may possess both endo- and exo-acting activities. The catalytic mechanism of PL10 enzymes need to be further studied.

### 2.6. Application of PpPel10a in Viscosity Reduction and Ramie Degumming

**Figure 9:**
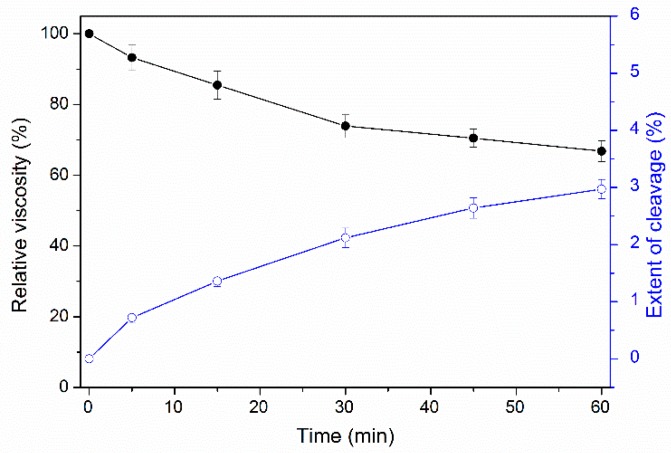
Reduction of PGA viscosity by PpPel10a (-●-) and galacturonoside bond cleavage (-○-).

The viscosity of a PGA solution decreased gradually during incubation at 50 °C for 1 h on addition of PpPel10a; the relative viscosity was reduced by 33.2% after 1 h of incubation (Figure 9). The reducing sugars released from PGA indicated that <3% of the galacturonosidic linkages were cleaved, which indicated that PpPel10a acts as an endo-pectate lyase toward PGA.

The ramie degumming effect of PpPel10a was studied. After treatment with 1 U/mL PpPel10a, 22.5 ± 0.68% of the weight of the ramie fibers was lost, whereas 10.1 ± 0.57% weight loss was obtained on treatment with 50 mM sodium glycine buffer, pH 9.0. In combined enzyme and chemical (sodium hydroxide) treatment, the weight loss was 26.3 ± 0.68%, which was higher than on chemical treatment alone (0.5% *w*/*v* NaOH, 19.9 ± 0.87%; 1% *w*/*v* NaOH, 25.9 ± 0.65%). Although the weight losses showed no significant difference between chemical-enzyme combination and 1% sodium hydroxide, the amount of sodium hydroxide used in the chemical-enzyme combination was 50% lower, which is better for the environment.

**Figure 10:**
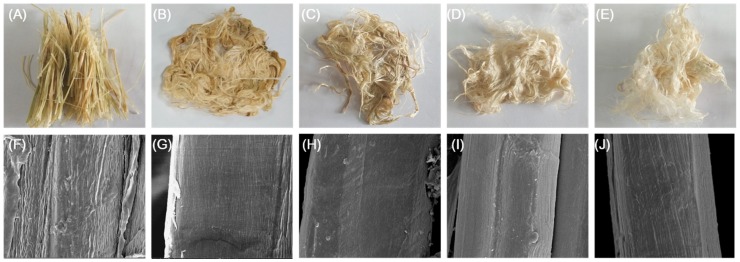
Ramie fibers treated with buffer (50 mM sodium glycine buffer, pH 9.0, negative control) (**A**,**F**); chemically (0.5% *w*/*v* sodium hydroxide (**B**,**G**), or 1% *w*/*v* sodium hydroxide (**C**,**H**)); enzymatically (1 U/mL PpPel10a) (**D**,**I**); or with enzyme-chemical combination (1 U/mL PpPel10a and 0.5% *w*/*v* sodium hydroxide) (**E**,**J**). Images **A**–**E**: extrinsic features of ramie fibers; images **F**–**J**: scanning electron microscopy images of single fibers (5000×).

The ramie textile treated by enzyme and enzyme plus chemical became much softer, smoother and whiter compared with that treated with 50 mM sodium glycine buffer (pH 9.0) or by sodium hydroxide alone, which is favorable for the textile industry. The textiles were observed by scanning electron microscopy (SEM). Figure 10 shows that the enzyme-treated and enzyme-chemical treated samples showed a smoother surface than the control group, which suggested that the gum-like material, i.e., the pectin, was removed. 

Recently, ramie degumming by some *Bacillus* spp. alkaline pectate lyases was reported [11,32]. Treatment with pectate lyases from *B. pumilus* DKS1, *B. subtilis* 7-3-3, and *B. pumilus* ATCC7061 resulted in 17%, 13.5% and 23.1% weight loss, respectively. The degumming efficiency of PpPel10a was higher than those of these pectate lyases. It is reported that the total gum content of ramie fibers is approximately 30–34% [14]. The reason of the incomplete degumming by PpPel10a may derive from the pH stability of PpPel10a at extreme alkaline environment. The pH of 0.5% NaOH in 20 mL of 50 mM sodium glycine buffer (pH 9.0) was determined to be ~12.5. The activity and stability of PpPel10a in this solution was studied. PpPel10a showed moderate activity at this pH, 62.5% of its activity retained at this pH. After incubated at this pH for 4 h at 50 °C, only 45.6% of its activity was detected. Therefore, the degumming efficiency needs to be further improved by improving the stability of PpPel10a or by using PpPel10a combined with other enzymes. 

Degumming of ramie fibers needs robust pectate lyases that are active and stable at alkaline pH and moderate temperature [10,11,12]. PpPel10a may have potential for use in this way because of its alkaline-tolerance and thermostability. In addition, PpPel10a was easily prepared since purification of the enzyme required only one step. Altogether, the enzyme-chemical combination degumming was more environmentally friendly than the traditional chemical degumming process. In the future, the degumming process will be further optimized.

## 3. Materials and Methods 

### 3.1. Bacterial Strains, Plasmid, and Substrates

*P. polymyxa* KF-1 was collected by our laboratory and deposited in the China Center for Type Culture Collection (CCTCC AB 2018146). *Escherichia coli* DH5α was used for gene cloning procedures. pET-28a(+) and *E. coli* BL21 (DE3) cells (Novagen, Madison, MI, USA) were used for recombinant enzyme production. Citrus pectin (P8030) and PGA (P8510) were purchased from Solarbio (Beijing, China). 

### 3.2. Screening of Pectate Lyase from P. polymyxa KF-1 by LC-MS/MS 

Strain *P. polymyxa* KF-1 was inoculated into 100 mL Luria-Bertani (LB) broth and shaken at 200 rpm at 30 °C for 36 h. After that, the supernatant of culture broth was obtained by centrifugation at 5000× *g* and 4 °C for 10 min. The supernatant was loaded onto a SP Sepharose fast-flow column (1.5 × 5 cm; GE Healthcare, Little Chalfont, Buckinghamshire, UK). The column was eluted by a stepwise gradient of 0–0.5 M sodium chloride (72 mL) in 25 mM Tris-HCl, pH 7.5. The pectate lyase activity of each fraction was measured with 0.2% PGA as substrate. The reaction was carried out in 50 mM sodium glycine buffer (pH 9.0) at 50 °C for 15 min. The absorbance increase at 235 nm was recorded. The extinction coefficient of unsaturated galacturonic acid at 235 nm is 4600 M^−1^ cm^−1^ [33]. One unit (U) of activity was defined as the amount of enzyme needed to produce 1 μmol/min unsaturated oligogalacturonide.

The chromatographic fraction with maximum pectate lyase activity was analyzed by Orbitrap Fusion Lumos LC-MS/MS (Thermo Fisher Scientific, Waltham, MA, USA) by Beijing Bio-Tech Pack Technology Company Ltd. (Beijing, China). Briefly, the proteins were in-solution digested by 100 ng/mL trypsin, the peptides were extracted by a mixture of trifluoroacetic acid/acetonitrile/distilled water (5/50/45, *v*/*v*/*v*), then separated using Acclaim PepMap RSLC C18 column (Thermo Fisher Scientific, Waltham, MA, USA). The separated peptides were analyzed by nanoLC-MS/MS [34]. The data obtained was searched against the UniProt nonredundant protein database for *P. polymyxa* using Proteome Discoverer 1.4 (Thermo Fisher Scientific, Waltham, MA, USA) [35].

### 3.3. Sequence Analysis of PL Family 10 Pectate Lyase

The peptides identified by LC-MS/MS analysis were analyzed by Protein BLAST (https://blast.ncbi.nlm.nih.gov/Blast.cgi). Amino acid sequence alignment was performed using MEGA version 6.06 (https://www.megasoftware.net/) [36]. Signal peptides were predicted using the SignalP 4.1 Server [25]. A structural model of the pectate lyase PpPel10a was generated using SWISS-MODEL with the family 10 polysaccharide lyase from *C. cellulosa* (PDB ID: 1GXN, identity = 44.79%) as the template [28,37]. 

### 3.4. Gene Cloning and Protein Expression

The pectate lyase gene *PpPel10a* (NCBI accession number WP_013370345.1) was amplified from genomic DNA of *P. polymyxa* KF-1 using primers 5′-CGGGATCCGAGCAGAATTTGACTGATGC-3′ and 5′-CCGCTCGAGTTATTGTGGCAACGGCTTGGACAG-3′. The PCR product was digested with *Bam*HI and *Xho*I, and ligated into *Bam*HI/*Xho*I-digested pET-28a(+). The ligation product was transformed into *E. coli* DH5α cells and the recombinant plasmid pET-28a-PpPel10a obtained was used to transform *E. coli* BL21 (DE3) cells [38]. The transformant was cultured in 200 mL LB medium supplemented with 30 μg/mL kanamycin [13]. Protein induction was initiated by the addition of 0.5 mM IPTG when the optical density of the culture at 600 nm reached 0.6. The culture was grown for another 10 h at 25 °C with shaking at 200 rpm [13].

Recombinant *E. coli* BL21 (DE3) cells were disrupted by sonication, and centrifuged at 12,000× *g* at 4 °C for 30 min to remove cell debris. The supernatant obtained (200 mL) was loaded onto a Ni-NTA agarose column (1 × 5 cm). The column was eluted with a linear gradient of imidazole (10–200 mM) in 25 mM Tris-HCl buffer, pH 7.5 [4]. Fractions with pectate lyase activity were collected and dialyzed into 25 mM Tris-HCl buffer (pH 8.0). Recombinant enzyme PpPel10a was analyzed by 10% SDS-PAGE [39]. The protein concentration was determined using BCA reagent [40].

### 3.5. Enzymatic Characterization of Pectate Lyase

The effect of pH on the activity of PpPel10a was determined at pH 2.0–11.0 using 0.2% PGA as the substrate. The reactions were carried out at 50 °C for 15 min. The effect of pH on stability was determined by incubating purified recombinant PpPel10a at 4 °C for 24 h at different pH, then the enzyme activity was assayed in standard conditions (50 °C, 15 min). The buffers used in this study were: pH 2.0–6.0, 50 mM sodium acetate buffer; pH 6.0–8.0, 50 mM phosphate buffer; pH 8.0–11.0, 50 mM sodium glycine buffer. The effect of temperature on enzyme activity was studied at 20–80 °C at pH 9.0 for 15 min. Thermostability of PpPel10a was studied by measuring residual pectate lyase activity after preincubating the enzyme at different temperatures for up to 1 h [41]. Then, the residual activities were determined at pH 9.0, 50 °C, over 15 min. 

The effects of metal ions or chemical compounds (1 and 5 mM) on pectate lyase activity were measured by preincubation of PpPel10a in different metal ion solutions or chemicals at 4 °C for 24 h. Then, the residual activities were measured using 0.2% PGA as the substrate at pH 9.0 and 50 °C for 15 min [13]. Enzyme without preincubation was defined as 100% active. The effect of Ca^2+^ on pectate lyase activity was determined from 0.1 to 5 mM Ca^2+^. The enzymatic reaction without the addition of Ca^2+^ was set as 100% activity. The kinetic parameters of PpPel10a were measured using 0.1–5.0 mg/mL PGA in standard assay conditions (pH 9.0, 50 °C, 15 min). Kinetic values K_m_, v_max_ and k_cat_ were calculated from Lineweaver-Burk plots [42].

### 3.6. Analysis of Lytic Products from Citrus Pectin on Degradation by PpPel10a

The change of molecular weight of citrus pectin after treatment with PpPel10a was analyzed by HPGPC using a TSK-gel G-3000PWXL column (7.8 × 300 mm; Tosoh, Tokyo, Japan) [43]. The column was calibrated with standard dextrans (1, 5, 12, 25 and 50 kDa) (Sigma-Aldrich, St. Louis, MO, USA). The unsaturated galacturonic acids released from citrus pectin by PpPel10a were analyzed by HPAEC with a pulsed amperometric detector [44]. The unsaturated galacturonic acids were identified by ESI-MS with the amaZon speed ion ETD trap (Bruker, Bremen, Germany) using negative electrospray as the ionization process [45].

### 3.7. Application of PpPel10a in Viscosity Reduction and Ramie Degumming

Purified PpPel10a (1.0 U) and PGA (0.2% *w*/*v*) were incubated in 10 mL of 50 mM sodium glycine buffer (pH 9.0) at 50 °C for up to 1 h. Viscosity was determined using a glass capillary viscometer (1833, Shenyi Glass, Shanghai, China) at timed intervals [24,46]. The viscosity of PGA without enzymatic treatment was considered as 100%. The extent of PGA cleavage was determined by measuring the released reducing sugars using the DNS method [46,47]. 

For ramie degumming, 2 g of ramie fibers were boiled in distilled water for 10 min, then treated by chemical (0.5% or 1% NaOH), enzyme (1 U/mL purified recombinant PpPel10a), or a chemical-enzyme combination (1 U/mL purified recombinant PpPel10a in 0.5% sodium hydroxide) in 20 mL of 50 mM sodium glycine buffer (pH 9.0). The mixtures were shaken at 50 °C and 100 rpm for 4 h. Then, the ramie fibers were washed twice and dried to constant weight. The weight loss was recorded, and the surface of the ramie fibers was observed by SEM [10,11,13]. Ramie fibers treated with 50 mM sodium glycine buffer (pH 9.0) were used as the negative control. The experiments were performed with five parallels. For ramie degumming, the activity and stability of PpPel10a in the chemical-enzyme combination treatment (0.5% NaOH in 20 mL of 50 mM sodium glycine buffer, pH 9.0) was studied using 0.2% PGA as the substrate. 

## 4. Conclusions

An alkaline pectate lyase was screened from *P. polymyxa* KF-1 and identified as belonging to PL family 10. The enzyme showed catalytic activity toward citrus pectin, producing unsaturated mono- and oligogalacturonates. The significant weight loss of ramie fibers treated with PpPel10a indicated the potential of this enzyme for application in ramie degumming. PpPel10a, which has good thermostability and pH stability, might be suitable for use in the textile industry.

## Figures and Tables

**Table 1:** Pectate lyases identified from *P. polymyxa* KF-1 by LC-MS/MS analysis.

Identified Peptide	Intensity (×10^10^)	Uniprot Accession No.	NCBI Accession No.	Signal Peptide ^1^	PL Family	Pfam Family	Predicted Mw (kDa) ^2^	Predicted *p*I
QPFDSDILDNTYR	1.6987	E3EEN8	WP_013370345.1	1–33	10	PF09492	45.24	9.41
SKDGVELGTFDNEATTTEIR								
EPGTVNITGGGAYHAYDK	1.4318	E3EDF5	WP_013369567.1	1–33	3	PF03211	24.619	9.19
TVVADPDTLGDGSQK								
VNMTLDNSDISNVK								
GADGSIQLGDFLK	0.4472	E3E7F9	WP_013373703.1	1–34	9	—	46.988	5.50
GLAASADDFVSLVPSITR								
GSDLIGSGTPSGNIGAR								
VIEIMNDLDLGWNEIPSAAK	0.054712	E0RB75	WP_013308307.1	1–32	1	PF00544	72.777	6.18
VKLELSGSEIK								

^1^ Signal peptides were predicted using the SignalP 4.1 server (http://www.cbs.dtu.dk/services/SignalP/) [25]. ^2^ Molecular weight and *p*I were predicted using the ExPASy Compute *p*I/Mw tool (https://web.expasy.org/compute_pi/) [26].

**Table 2:** Purification of *P. polymyxa* KF-1 Pel10a and of recombinant PpPel10a from *E. coli*.

Procedure	Volume (mL)	Total Activity (U)	Total Protein (mg)	Specific Activity (U/mg)	Yield (%)	Purification
Purification						
Fermentation broth	200	2762.5	85	32.5	100.0	1.0
SP Sepharose FF column	9	1066.85	9.5	112.3	38.6	3.5
Heteroexpression						
Crude enzyme extract	200	2,2766.4	216	105.4	100	1.0
Ni-NTA column	10	9889	34.1	290	43.4	2.7

**Table 3:** Effect of metal ions and chemical reagents on the enzyme activity of PpPel10a.

Metal Ion or Chemical	Relative Activity (%)	
	1 mM	5 mM
NaCl	99.3 ± 1.9	86.7 ± 3.25
KCl	90.2 ± 2.0	79.0 ± 0.55
CaCl_2_	195.5 ± 2.65	176.6 ± 3.2
MgCl_2_	113.7 ± 0.55	94.5 ± 4.65
FeSO_4_	65.1 ± 1.5	54.5 ± 0.65
EDTA	89.3 ± 0.15	63.1 ± 0.45
ZnCl_2_	115.2 ± 0.95	88.0 ± 1.55
AlCl_3_	119.6 ± 0.35	106.1 ± 3.1
CoCl_2_	72.2 ± 3.4	51.4 ± 2.4
SDS	61.7 ± 1.95	37.7 ± 0.15
Tween-20	108.0 ± 1.0	122.5 ± 0.55
Triton X-100	8.5 ± 0.75	4.3 ± 0.15

## Supplementary Materials

The following are available online, Figure S1: Measurement of *P. polymyxa* KF-1 pectate lyase activity during liquid culture, Figure S2: Structural model of PpPel10a generated by SWISS-MODEL using pectate lyase from *C. cellulosa* (PDB ID: 1GXN, identity = 44.79%) as the template.
