# Supplementary material for: Screening of a Novel Polysaccharide Lyase Family 10 Pectate Lyase from Paenibacillus polymyxa KF-1: Cloning, Expression and Characterization
Source: Molecules. 2018 Oct 26;23(11):2774. doi: 10.3390/molecules23112774 (PMC6278402; doi:10.3390/molecules23112774)
Supplement: Supplementary file 1 [file molecules-23-02774-s001.pdf]

*Supplementary file*

# Screening of a novel polysaccharide lyase family 10 pectate lyase from *Paenibacillus polymyxa* KF-1: cloning, expression and characterization

**Yan Zhao[1, #], Ye Yuan[2, #], Xinyu Zhang[1], Yumei Li[1], Qiang Li[1], Yifa Zhou[2], Juan Gao[1, *]**

[1] School of Biological Science and Technology, University of Jinan, Jinan 250022, PR China

[2] School of Life Sciences, Northeast Normal University, Changchun 130024, PR China

* Correspondence: bio_gaoj@ujn.edu.cn; Tel.: +86-531-89736825

# These authors contributed equally to this work.

**Table S1**. Proteins identified from *P. polymyxa* KF-1 by LC-MS/MS analysis

| Identified peptide | Intensity (× $10^{10}$) | Uniprot accession No. | NCBI accession No. | Signal peptide[1] | PL Family | Pfam family | Predicted Mw (kDa)[2] | Predicted *p*I |
|---|---|---|---|---|---|---|---|---|
| QPFDSDILDNTYR<br>SKDGVELGTFDNEATTTEIR | 1. 6987 | E3EEN8 | WP_013370345.1 | 1-33 | 10 | PF09492 | 45.24 | 9.41 |
| EPGTVNITGGGAYHAYDK<br>TVVADPDTLGDGSQK<br>VNMTLDNSDISNVK | 1.4318 | E3EDF5 | WP_013369567.1 | 1-33 | 3 | PF03211 | 24.619 | 9.19 |
| GADGSIQLGDFLK<br>GLAASADDFVSLVPSITR<br>GSDLIGSGTPSGNIGAR | 0.4472 | E3E7F9 | WP_013373703.1 | 1-34 | 9 | — | 46.988 | 5.50 |
| VIEIMNDLDLGWNEIPSAAK<br>VKLELSGSEIK | 0.054712 | E0RB75 | WP_013308307.1 | 1-32 | 1 | PF00544 | 72.777 | 6.18 |

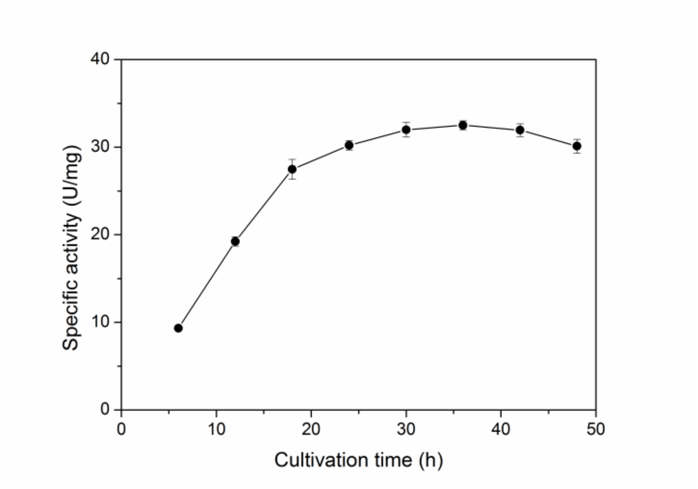


**Figure S1.** Measurement of pectate lyase activity of *P. polymyxa* KF-1 during liquid culture.

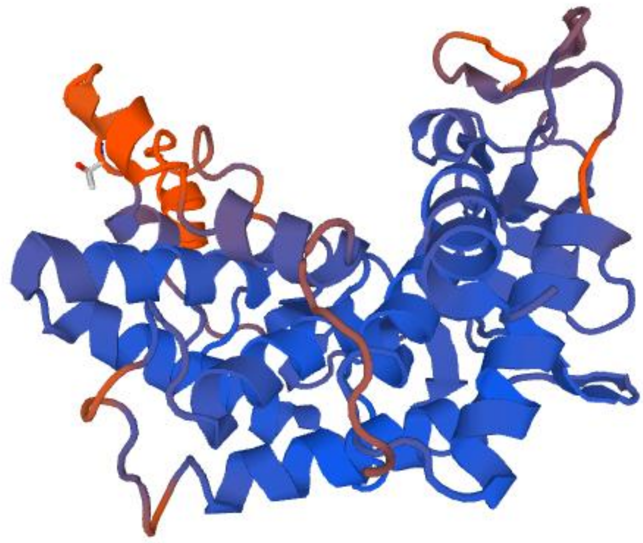

**Figure S2.** Structure modeling of PpPel10a by Swiss-MODEL using pectate lyase from *C. cellulosa* (PDB ID: 1GXN, identity = 44.79%) as a template.
